# Au- or Ag-Decorated ZnO-Rod/rGO Nanocomposite with Enhanced Room-Temperature NO_2_-Sensing Performance

**DOI:** 10.3390/nano13162370

**Published:** 2023-08-18

**Authors:** Ke Huang, Junfeng Lu, Donglin Li, Xianjia Chen, Dingfeng Jin, Hongxiao Jin

**Affiliations:** College of Materials Science and Engineering, China Jiliang University, Hangzhou 310018, China

**Keywords:** Au nanoparticles, Ag nanoparticles, rGO, ZnO rods, p-n junction, synergistic effect

## Abstract

To improve the gas sensitivity of reduced oxide graphene (rGO)-based NO_2_ room-temperature sensors, different contents (0–3 wt%) of rGO, ZnO rods, and noble metal nanoparticles (Au or Ag NPs) were synthesized to construct ternary hybrids that combine the advantages of each component. The prepared ZnO rods had a diameter of around 200 nm and a length of about 2 μm. Au or Ag NPs with diameters of 20–30 nm were loaded on the ZnO-rod/rGO hybrid. It was found that rGO simply connects the monodispersed ZnO rods and does not change the morphology of ZnO rods. In addition, the rod-like ZnO prevents rGO stacking and makes nanocomposite-based ZnO/rGO achieve a porous structure, which facilitates the diffusion of gas molecules. The sensors’ gas-sensing properties for NO_2_ were evaluated. The results reveal that Ag@ZnO rods-2% rGO and Au@ZnO rods-2% rGO perform better in low concentrations of NO_2_ gas, with greater response and shorter recovery time at the ambient temperature. The response and recovery times with 15 ppm NO_2_ were 132 s, 139 s and 108 s, 120 s, and the sensitivity values were 2.26 and 2.87, respectively. The synergistic impact of ZnO and Au (Ag) doping was proposed to explain the improved gas sensing. The p-n junction formed on the ZnO and rGO interface and the catalytic effects of Au (Ag) NPs are the main reasons for the enhanced sensitivity of Au (Ag)@ZnO rods-2% rGO.

## 1. Introduction

With the acceleration of modern industrial processes, industrial emissions are intensifying, and many harmful gases are discharged straight into the atmosphere without being decontaminated [1,2]. Among the toxic gases, nitrogen oxide (NO_2_) receives special attention because of its deadly toxicity. It is a typical pollution emission produced by the burning of fossil fuels in power plants, automobile engines, and other stationary sources, and is damaging to the environment and human health [3,4]. NO_2_ must be detected in real time in order to adequately monitor air quality and ensure public safety. The development of NO_2_ gas sensors with high sensitivity, low detection limits, and excellent selectivity is crucial [5,6,7,8].

To detect harmful gas leaks and track air pollution, semiconductor-based gas sensors are widely utilized. Due to their distinct structures and special surface area, materials including TiO_2_, ZnO, SnO_2_, and CuO have been widely employed to make gas sensors [9,10,11]. ZnO attracts much attention among these due to its consistent physicochemical characteristics, controllable shape and microstructure, the absence of toxicity, and favorable gas-sensitive features. After SnO_2_, it is the most significant semiconductor metal oxide with a direct broad band gap (3.37 ev) [12,13]. However, a great deal of reported gas sensors based on metal oxide semiconductors do not perform sufficiently in practical uses at ambient temperatures because they require a sufficient amount of reaction activation energy between the gas being detected and oxygen ions that are adsorbed on the surface. When the temperature is more than 150 °C, the reaction activation energy is typically delivered in appropriate amounts; however, a greater working temperature increases the consumption of energy and economical costs, which restrict the growth and application of gas sensors [14,15,16].

Making room-temperature sensing materials by modifying the semiconductor characteristics by adding reduced graphene oxide (rGO) is a potential method. rGO interacts well with target gases because it has a lot of functional groups, dangling bonds, and defects [17,18,19]. To obtain the desired room-temperature NO_2_ sensor with high sensitivity, quick response, and fast recovery, and combining the advantages of metal oxides and rGO, a gas sensor was constructed. A heterojunction is formed between metal oxides and rGO [20,21], enabling NO_2_ monitoring to be sensitive and quick at moderate temperatures of operation.

Beyond this, an extensive amount of theoretical research and experimental work has demonstrated that one of the best strategies to improve rGO-based gas sensors is to decorate graphene with noble metal nanoparticles [22,23]. Noble metal nanoparticles are added, increasing the material’s surface area specifically and its active adsorption site [24,25]. Additionally, this somewhat modifies the electrical structure and carrier concentration of graphene. Noble metal nanoparticles, like Ag, Au, and Pt NPs, have a particular catalytic effect that can lower the activation energy of the interaction between a material’s surface and a target gas, improving the material’s reactivity to the target gas. Gautam M et al. modified CVD graphene using platinum and gold nanoparticles to improve graphene’s ability to sense VOC chemicals like acetic acid and ethanol [26].

Herein, the Au or Ag NPs are mixed with ZnO/rGO hybrids to obtain Au@ZnO rods-rGO and Ag@ZnO rods-rGO through one-pot hydrothermal synthesis. The nanocomposites exhibit good NO_2_-sensing properties at room temperature. The materials’ superior gas-sensing abilities result from an improved synergistic interaction between the Ag (Au) NPs, ZnO rods, and rGO, which may provide a new solution for room-temperature and selective NO_2_ gas sensing.

## 2. Materials and Methods

### 2.1. Chemicals

H_2_SO_4_ (98 wt%), HCl (36 wt%), NaNO_3_, Na_3_C_6_H_5_O_7_·2H_2_O (sodium citrate), N_2_H_4_·H_2_O (50 wt%), glucose, and AgNO_3_ were obtained from Zhejiang Zhong liang Chemical Reagent Co., Ltd., Zhejiang, China. Graphite powder was received from Nanjing Xianfeng Nanomaterials Technology Co., Ltd., Nanjing, China. Zn(NO_3_)_2_·6H_2_O, KMnO_4_, and H_2_O_2_ (30 wt%) were purchased from Shanghai Lingfeng Chemical Reagent Co., Ltd., Shanghai, China. HAuCl_4_·3H_2_O and ethanol were obtained from Aladdin Reagent Co., Ltd., Jiangsu, China. All chemicals were of analytical quality and were utilized without additional purification. A Millipore system was utilized to clean the water before usage.

### 2.2. Preparation of Sensing Materials

Graphene oxide: GO nanosheets were produced from graphite powder using Hummer’s approach. First, 69 mL of sulfuric acid were used to dissolve 3.0 g of graphite powder, and the mixture was rapidly agitated for 10 min. To create a homogenous solution, 1.5 g of sodium nitrate was added to the mixture, and it was maintained at 0 °C throughout this procedure. The mixture was then given 9.0 g of potassium permanganate, forcefully agitated for 3 h, and then constantly swirled for an additional 6 h at 35 °C. Following that, 550 mL of distilled water and 20 mL of H_2_O_2_ were added and mixed constantly for 2 h. The samples were then repeatedly cleaned with distilled water until the pH was 7. The material was dialyzed for two weeks to entirely eliminate metal ions. From the freeze-dried GO powder, a graphene oxide/water dispersion (5 mg/mL) was created for further application.

ZnO rods: A total of 5.955 g of Zn(NO_3_)_2_·6H_2_O was added to dissolve in 150 mL of water with stirring, followed by 8 g of N_2_H_4_·H_2_O. The solution was then placed in an autoclave coated with Teflon and heat-treated for 24 h at 200 °C. After cooling to ambient temperature, it was centrifuged and treated many times with distilled water and ethanol. The ZnO rod powder was made by drying the powder at 60 °C for 12 h.

Au sol: A total of 100 mL of sodium citrate solution (17 mmol/L) was heated to boil. Then, 1.5 mL of HAuCl_4_·3H_2_O (4 g/L) solution was added under vigorous stirring. The mixture was boiled for another 10–15 min and then cooled to room temperature to obtain gold sol.

Ag sol: A total of 0.072 g of AgNO_3_ was dissolved in 400 mL water and heated to boil under vigorous stirring. Then, 8 mL solution of 1 wt% sodium citrate was added. The solution was kept boiling for about 1 h to obtain the Ag colloid.

ZnO rods/rGO: A homogeneous ZnO/GO dispersion with 1%, 2%, and 3% GO in mass ratio was created by dispersing 1 g of ZnO powder rods in 30 mL of water, then sonicating the mixture for 30 min. After rapidly stirring the mixture for 3 min, the desired amounts of graphene oxide dispersion were added to the ZnO dispersion. The homogeneous solution was then moved to a 50 mL autoclave lined with Teflon and heated at 180 °C for 9 h. The product was rinsed with water and freeze-dried after it had cooled to room temperature. The collected samples were designated as ZnO rods-1% rGO, ZnO rods-2% rGO, and ZnO rods-3% rGO based on their mass ratio. To validate the hydrothermal reduction of GO to rGO, pure rGO was produced under the same conditions.

Au@ZnO rods-2% rGO and Ag@ZnO rods-2% rGO: A total of 1 g of ZnO powder rods was placed in 28 mL of water, followed by the desired amount of GO (2 wt%), and the resulting mixture was then sonicated for 30 min. Then, with vigorous stirring, 2 mL of the above Au or Ag sol was added drop by drop. The mixture was placed in an autoclave and heated at 180 degrees for 9 h. The samples were washed with water multiple times before being freeze-dried to eliminate extra contaminants. The samples were labeled as Au@ZnO rods-2% rGO and Ag@ZnO rods-2% rGO.

### 2.3. Characterizations

To examine the phase and crystallinity of the composite materials, X-ray diffraction (XRD) patterns were recorded on a diffractometer (D8-ADVANCE, Bruker, Leipzig, Germany) with Cu K radiation (=1.5418). Field-emission scanning electron microscopy (FESEM, S-4800, Hitachi Ltd., Chiyoda, Japan) and high-resolution transmission electron microscopy (TEM, JEM-2100, JEOL, Tokyo, Japan) were used to evaluate the morphology and microstructures of the as-prepared samples. A spectrometer with an Al K X-ray source (Thermo ESCALAB 250 Xi, Waltham, MA, USA) was used for X-ray photoelectron spectroscopy (XPS). A nitrogen adsorption analyzer (Micromeritics ASAP 2020, Merrick, Norcross, GA, USA) was used to estimate the BET surface areas of the materials. A laser confocal microscope Raman spectrometer (HORIBA, LabRAM HR, Paris, France) was used to record Raman spectra.

### 2.4. Gas Sensor Fabrication

A total of 0.5 mL of ethanol was added to 5 mg of the material powder, and the mixture was thoroughly crushed to create a paste. The coating substance was then uniformly applied to an interdigital electrode, and it was left to dry overnight. The sensing film (~200 nm in thickness) was then put in a muffle furnace and kept at 200 °C for 6 h. On a CGS-4TPS system (made by Beijing, China’s Alite Technology Co., Ltd., Beijing, China), the gas-sensing capability was assessed. Temperatures of 25 °C were used for the measurements. Ra/Rg defines the sensitivity (S) of the sensor, where Ra is the initial resistance in dry air and Rg is the resistance of the sensitive film upon exposure to NO_2_ environment. The reaction time is defined as the time necessary to attain 90% of the total response, whereas the recovery time is the time required to restore 90% of the baseline value.

## 3. Results

### 3.1. Characterization

**Figure 1:**
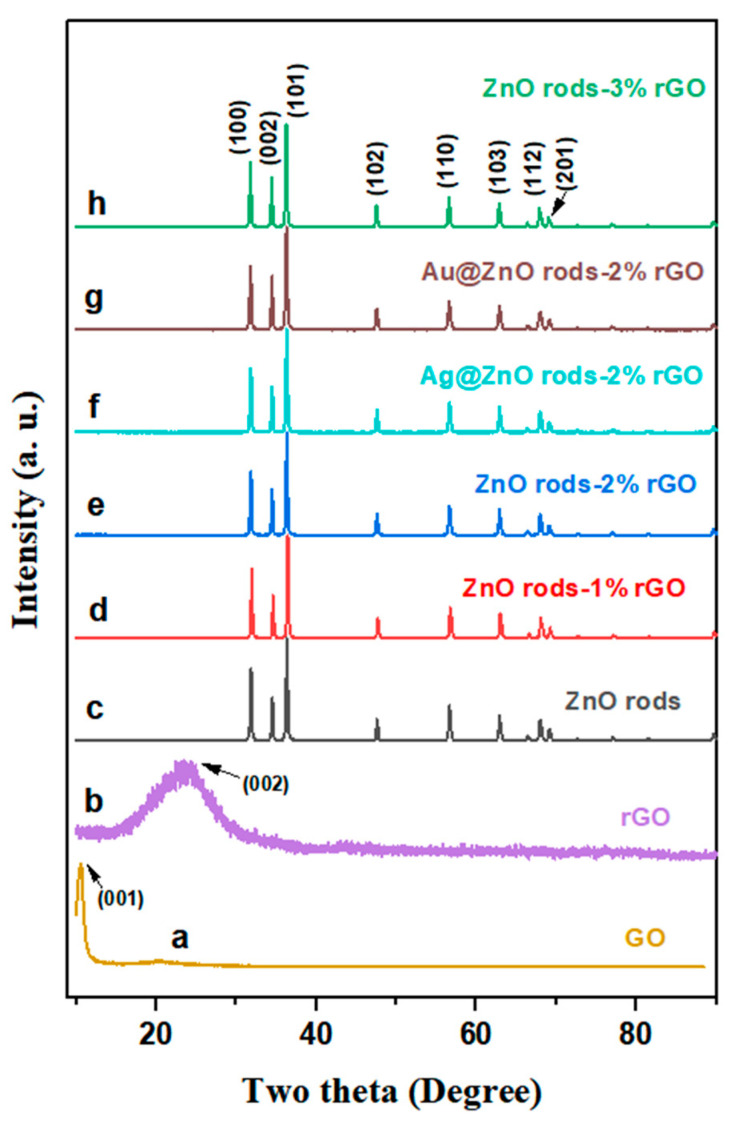
XRD patterns of GO, rGO, ZnO, ZnO rods-1% rGO, ZnO rods-2% rGO, Ag@ZnO rods-2% rGO, Au@ZnO rods-2% rGO, and ZnO rods-3% rGO.

Powder X-ray diffraction (XRD) was used to investigate the materials’ structure and crystalline phase. The diffraction peak at 2Theta = 10.5 degrees (Figure 1, line a) corresponds to the (001) GO reflection. After the hydrothermal reduction of GO to rGO, a wide peak can be observed at 25 degrees (Figure 1, line b), matching the graphene planes (002). The fluctuation of the peaks demonstrates that GO was successfully reduced under the same hydrothermal conditions as those under which the Zn/rGO or Au (Ag) @Zn/rGO hybrids were being synthesized. All the peaks in the ZnO rods and their hybrids (Figure 1, lines c–h) excellently fitted the hexagonal wurtzite phase of ZnO (JCPDS no.79-206), and no impurity phase was detected. The peaks that appeared at 2θ = 31.84, 34.50, 36.32, 47.60, 56.64, 62.90, 66.42, 67.96, and 69.12 degrees can be indexed to the (100), (002), (101), (102), (110), (103), (200), (112), and (004) facets of ZnO, respectively. However, no distinct diffraction peaks of rGO (Figure 1, lines d–h), Ag NPs (Figure 1, line f), or Au NPs (Figure 1, line g) were observed of the hybrids. This is related to the small content of rGO and Ag and Au NPs, and also because of their weak peak intensities [27,28].

**Figure 2:**
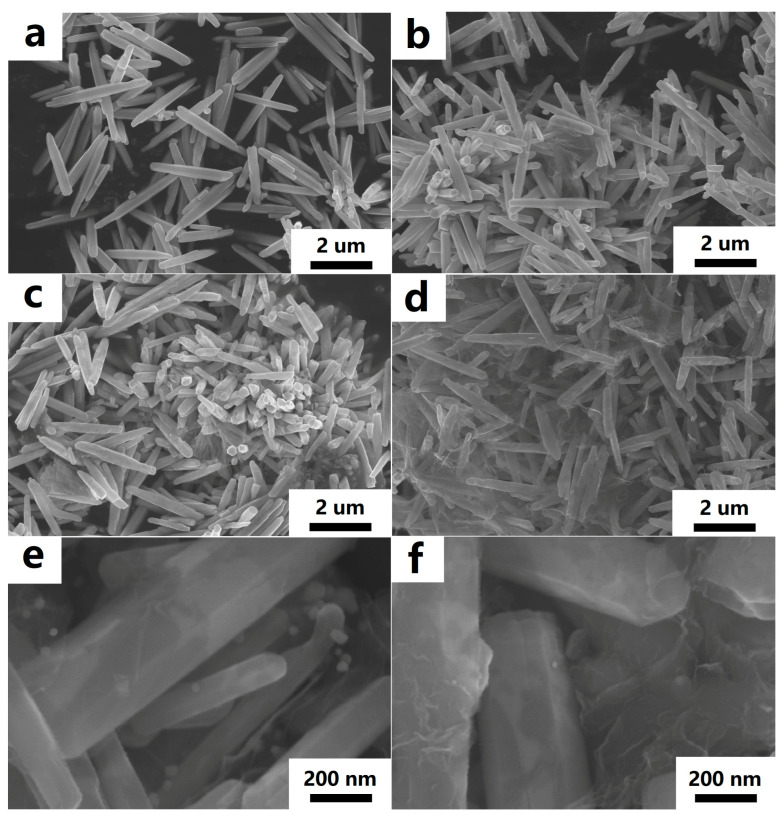
FESEM images of ZnO rods (**a**), ZnO rods-1% rGO (**b**), ZnO rods-2% rGO (**c**), ZnO rods-3% rGO (**d**), Ag@ZnO rods-2% rGO (**e**), and Au@ZnO rods-2% rGO (**f**).

**Figure 3:**
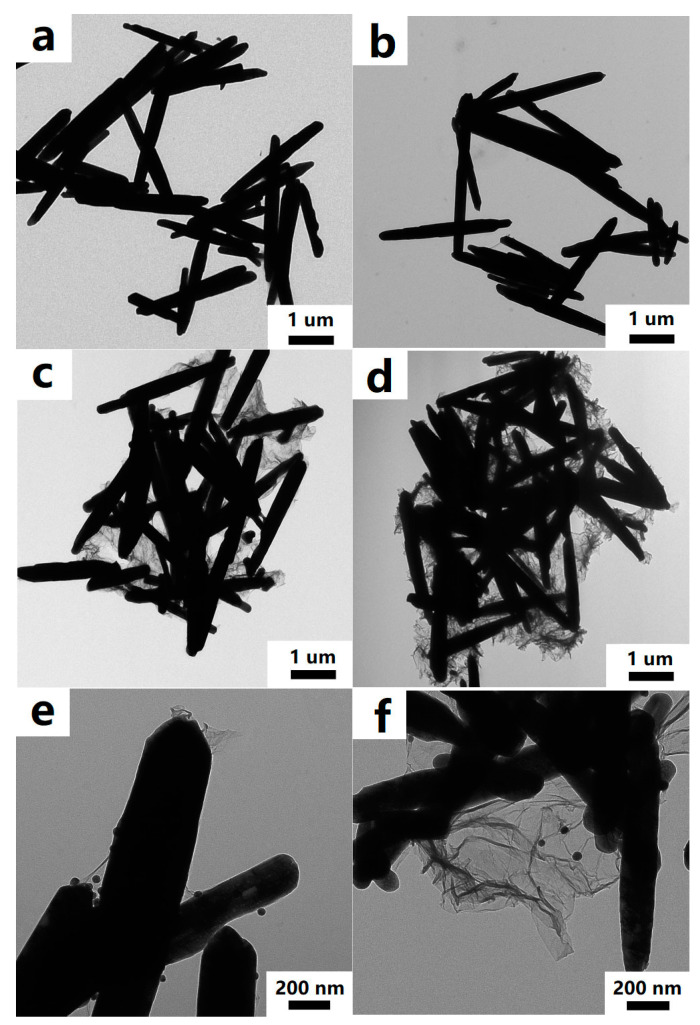
TEM images of ZnO rods (**a**), ZnO rods-1% rGO (**b**), ZnO rods-2% rGO (**c**), ZnO rods-3% rGO (**d**), Ag@ZnO rods-2% rGO (**e**), and Au@ZnO rods-2% rGO (**f**).

**Figure 4:**
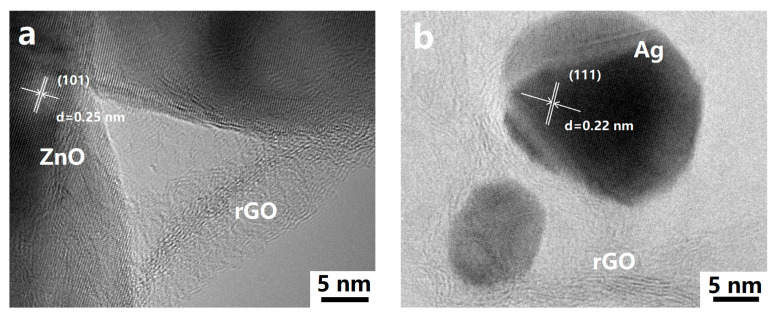
Typical HRTEM images of ZnO rods-2% rGO (**a**) and Ag@ZnO rods-2% rGO (**b**).

The morphology and microstructure of the ZnO rods-rGO were investigated using SEM and TEM. The prepared ZnO rods were about 200 nm in diameter and 5 μm in length (Figure 2a and Figure 3a), and there was a certain degree of connection and agglomeration between the ZnO rods. The original rod morphology was maintained after rGO was added to form a hybrid with ZnO (Figure 2b–d and Figure 3b–d). It was found that rGO simply covered the surface of ZnO and connected the ZnO rods like a “bridge”. As the concentration of rGO increases, rGO connects and encapsulates ZnO rods to a greater extent and the degree of ZnO aggregation increases. The introduction of Ag (Figure 2e and Figure 3e) or Au NPs (Figure 2f and Figure 3f) did not have a significant effect on the ZnO nanorod morphology during the hydrothermal synthesis. We observed relatively small Ag or Au NPs with diameters of 10–30 nm dispersed on flexible nets like rGO sheets. The selected HRTEM images show the interface between ZnO (Figure 4a) or Ag NPs (Figure 4b) and rGO. The lattice fringes with a d-spacing of 0.25 nm correspond to the (101) plane of the hexagonal wurtzite phase of ZnO (Figure 4a). The lattice fringes with a d-spacing of 0.22 nm (Figure 4b) correspond to the hkl(111) planes of the FCC structure of silver nanoparticles (JCPDS card: 04-0783).

**Figure 5:**
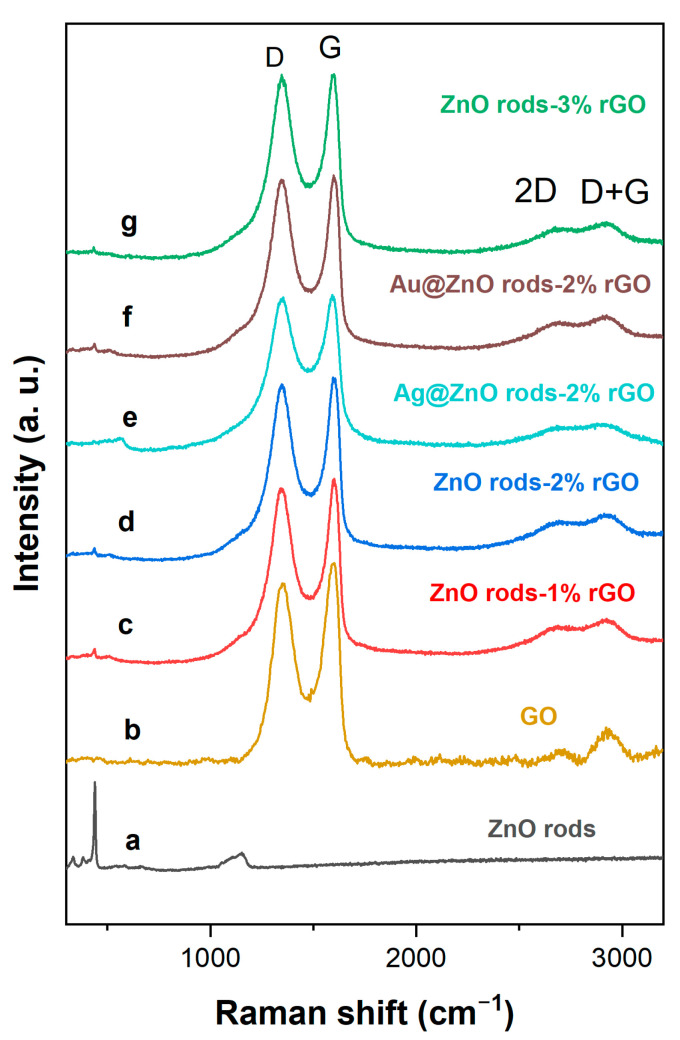
Raman spectra of ZnO rods (a), GO (b), ZnO rods-1% rGO (c), ZnO rods-2% rGO (d), Ag@ZnO rods-2% rGO (e), Au@ZnO rods-2% rGO (f), and ZnO rods-3% rGO (g).

The phase compositions of the materials were further studied through Raman spectroscopy (Figure 5). A weak peak at 439.8 cm^−1^ corresponded to the E_2_ mode of the ZnO rods. All of the hybrids have two intense peaks that correlate to the D and G bands, respectively. The D band at 1350 cm^−1^ is caused by the breathing mode of A_1g_ symmetry K-point phonons along the outside edges of graphite structures, which increases along with rising defect density (local defects or disorder). Even if the quantity of graphene is relatively small, the G band of approximately 1580 cm^−1^ conforms to the E_2g_ mode of sp^2^-hybridized carbon atoms (stacking nature) of the rGO species. The relative intensity of the D band to the G band (I_D_/I_G_) is commonly used as a metric to assess the disordered nature and defect extent of various carbon materials; that is, the greater the ID/IG, the greater the disorder in the carbon materials. The I_D_/I_G_ ratios of the ZnO rods-rGO are 0.95, 0.97, and 1.00, respectively, for the ZnO rods-1% rGO, ZnO rods-2% rGO, and ZnO rods-3% rGO, and all three are higher than that of GO (0.88) [29,30,31]. The presence of second-order D (2D) bands (~2667 cm^−1^) and D+G bands (~2918 cm^−1^), both of which belong to rGO, was also seen in the rGO and ZnO rods-rGO, confirming the creation of rGO during the synthesis. Furthermore, the I_D_/I_G_ ratios of Ag@ZnO rods-2% rGO and Au@ZnO rods-2% rGO are 0.997 and 0.975, respectively. This may be because the introduction of Ag or Au NPs leads to decreased disorder in graphene. Besides the above-mentioned points, the peak shift may be related to increased interaction between rGO and ZnO as the proportion of rGO increases [32,33].

**Figure 6:**
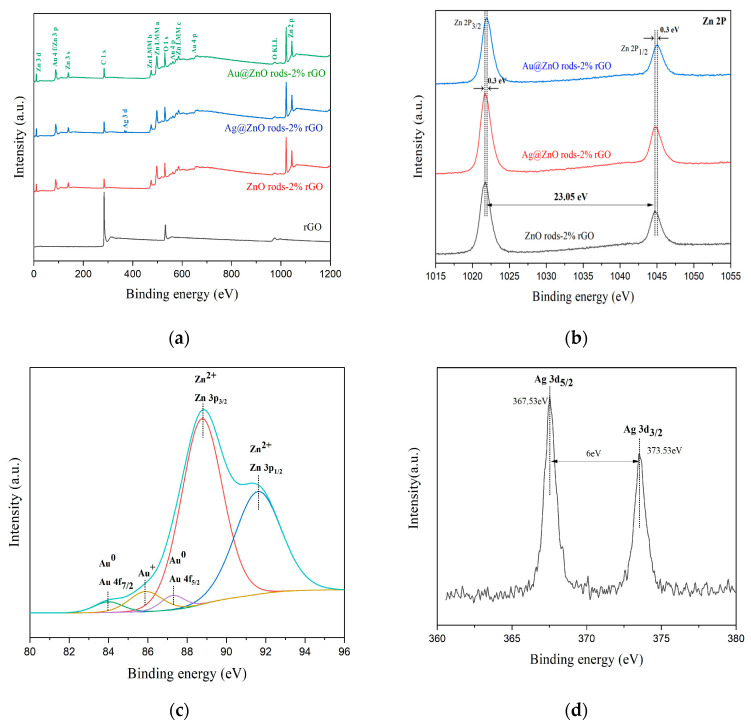

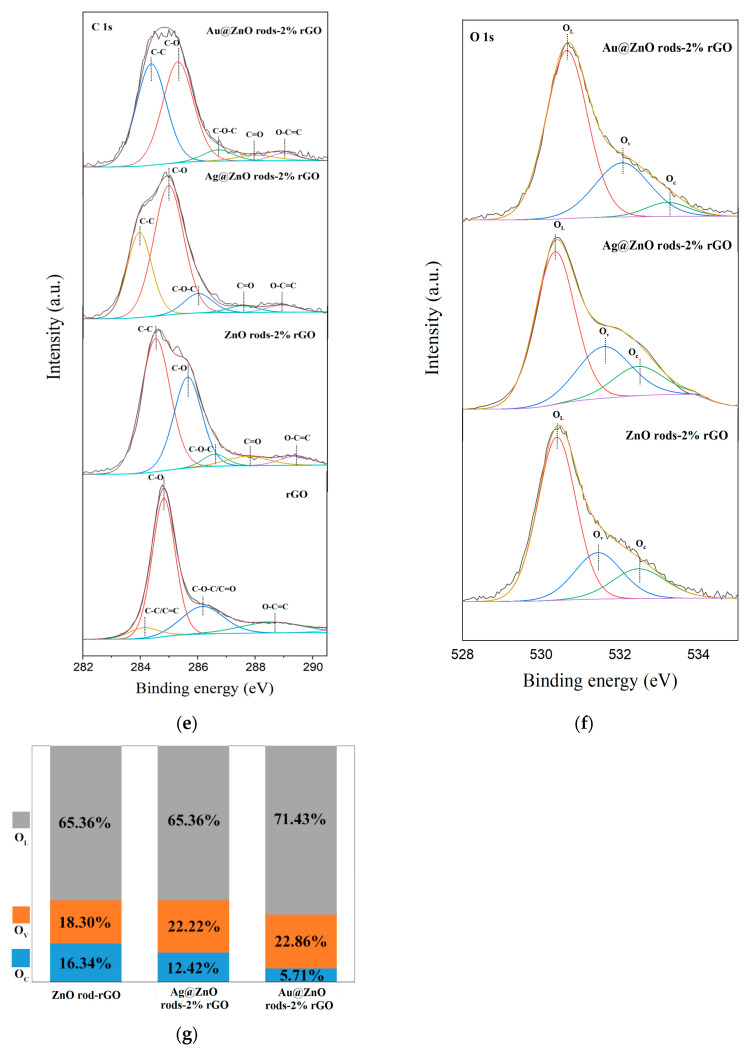
XPS spectra of rGO, ZnO rods-2% rGO, Au@ZnO rods-2% rGO, and Ag@ZnO rods-2% rGO: (**a**) survey spectrum, (**b**) Zn 2p region, (**c**) Au 4f region, (**d**) Ag 3d region, (**e**) C 1s region, (**f**) O 1s region, and (**g**) the percentages of the different oxygen species.

Using X-ray photoelectron spectroscopy, it is possible to determine the chemical and elemental composition of rGO, ZnO rods-2% rGO, Au@ZnO rods-2% rGO, and Ag@ZnO rods-2% rGO. The survey spectra of the materials (Figure 6a) show the peaks related to Zn, Au, Ag, O, and C elements. High-resolution spectra of Zn, Au, Ag, C, and O are shown in Figure 6b–f. (1) Zn (Figure 6b): With binding energies of 1021.7 eV and 1044.5 eV, respectively, two symmetrical peaks are seen in ZnO rods-2% rGO and Ag@ZnO rods-2% rGO that correspond to Zn 2p_3/2_ and Zn 2p_1/2_. Additionally, the separation between two peaks is around 23.1 eV, showing that Zn^2+^ is in a normal oxidation valence state in ZnO crystals. The Zn^2+^ binding energy peaks of Au-adorned ZnO/rGO were observed to be displaced (0.3 eV) in comparison with Zn 2p_1/2_ and Zn 2p_3/2_ of the other two samples. The changes demonstrate that there is electrical interaction between Au NPs and ZnO rods-2% rGO. (2) Au (Figure 6c): The Au 4f area and the Zn 3p region have a relative overlap. After deconvoluting the Au 4f area into Au 4f peaks with a Zn 3p doublet envelope, the chemical states of the Au 4f for the Au@ZnO rods-2% rGO sample were obtained. The metallic Au^0^ is primarily responsible for the Au 4f peaks at 83.9 and 87.5 eV, which correspond to the Au 4f_7/2_ and Au 4f_5/2_ core levels, respectively. Au+ from the Au 4f_7/2_ energy range is responsible for the other peak seen at 85.8 eV. When compared with bulk metallic gold, Au 4f’s observed binding energy is lower (84.2 eV and 87.8 eV). Due to the strong electrical contact between Au NPs and ZnO, the electron transfer from ZnO to the Au interface is responsible for this binding energy change. (3) Ag (Figure 6d): For Ag, the binding energy peaks are at 367.5 eV and 373.5 eV, and are attributed to Ag 3d_5/2_ and Ag 3d_3/2_, respectively. The silver on the surface of Ag@ZnO rods-2% rGO is metallic silver rather than oxidized silver, as evidenced by the peak separation of 6 eV between these two peaks. (4) C (Figure 6e): The narrow scan spectrum of the C 1s peak demonstrates that the C 1s spectrum can be divided into five peaks at 289.4, 287.8, 286.6, 285.6, and 284.5 eV, which correspond to the O-C=C, C=O, C-O-C, C-O, and C-C/C=C (attributed to the sp^2^/sp^3^ carbon atom) functional groups of rGO, respectively. After Au or Ag modification, compared with rGO, the characteristic peak of the C-C bond increased whereas those of the C-O, C=O, and O-C=O bonds decreased, suggesting removal of oxygen-containing functional groups. The ZnO rods-2% rGO sample exhibits the highest C-C bond ratio among the materials. (5) O (Figure 6f): The O 1s XPS peak shown in Figure 3f can be decomposed into three Gaussian components at ~532.5 eV (O_C_), 531.4 eV (O_V_), and 530.4 eV (O_L_). O_C_ can be indexed to chemisorbed oxygen species, O_V_ is caused by oxygen-containing particles that partially fill the oxygen-deficient zone caused by oxygen vacancies, and O_L_ is lattice oxygen in the wurtzite structure of hexagonal ZnO. The percentages of the different oxygen species for the four sensing materials are shown in Figure 6g; the O_C_ component of ZnO rods-2% rGO is relatively higher than those of Ag@ZnO rods-2% rGO and Au@ZnO rods-2% rGO. The higher percentages of O_C_ contribute to the performance of gas-sensing properties.

**Figure 7:**
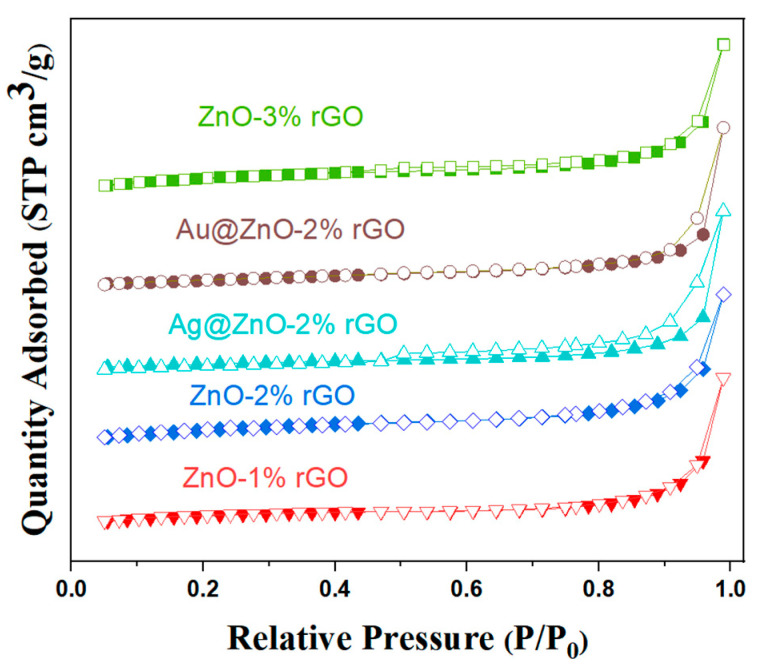
Nitrogen adsorption–desorption isotherms of ZnO rods-1% rGO, ZnO rods-2% rGO, Ag@ZnO rods-2% rGO, and Au@ZnO rods-2% rGO.

Figure 7 depicts the physical nitrogen adsorption–desorption curves of the samples. The adsorption isotherms of the samples in the figure are all type IV according to the IUPAC classification. The BET surface areas of ZnO rods-1% rGO, ZnO rods-2% rGO, ZnO rods-3% rGO, Ag@ZnO rods-2% rGO, and Au@ZnO rods-2% rGO were 5.515, 11.13, 13.30, 13.88, and 10.76 m^2^/g, respectively. All the samples showed increased surface areas compared with pure ZnO rods whose BET surface area was only 0.2712 m^2^/g. The specific surface area of the composites increases as the rGO doping ratio increases, while the addition of Ag or Au NPs has minimal influence on the surface areas. The ZnO rods prevent rGO agglomeration during synthesis, resulting in the formation of hybrids with high surface areas and rich pores that promote both the adsorption and desorption of target gas molecules on the surfaces and enhance the response and recovery performance of gas-sensing materials.

### 3.2. Gas-Sensing Properties

**Figure 8:**
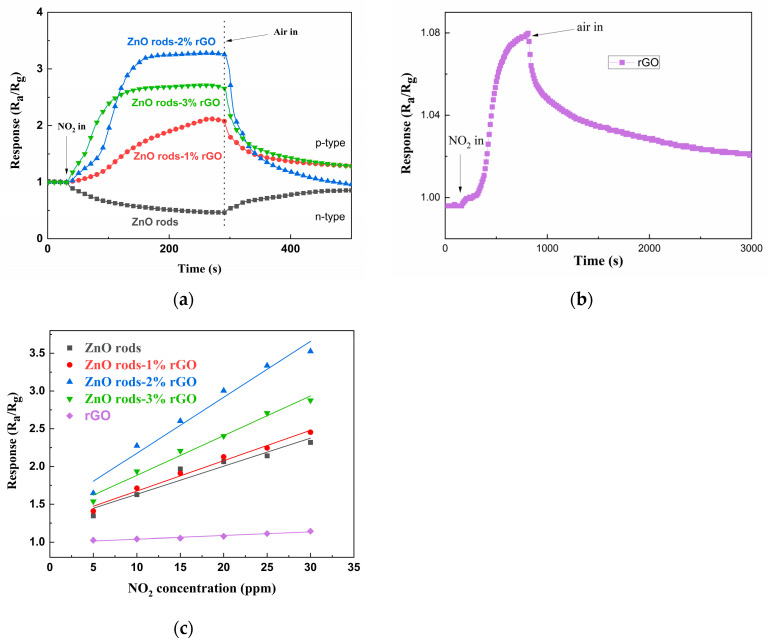
The dynamic response curves of ZnO rods, ZnO rods-1% rGO, ZnO rods-2% rGO, and ZnO rods-3% rGO to 25 ppm NO_2_ (**a**); the dynamic response curves of rGO to 25 ppm NO_2_ (**b**); the fitting curves of ZnO rods, ZnO rods-1% rGO, ZnO rods-2% rGO, ZnO rods-3% rGO, and rGO to 5–30 ppm NO_2_ at room temperature (**c**).

At room temperature, we initially investigated the detecting characteristics of ZnO rods, ZnO rods-1% rGO, ZnO rods-2% rGO, and ZnO rods-3% rGO samples for 25 ppm of NO_2_. The dynamic response–recovery curves (Figure 8a,b) demonstrate that the ZnO rod sensor had an n-type response with increasing resistance to NO_2_, but the rGO and ZnO rods-rGO sensors had a p-type response to NO_2_. Previously, we observed that the doping ratio depended on the sensing type of ZnO rods-rGO hybrid materials [34,35,36]. Under low rGO doping (<1%), temperature- and NO_2_ concentration-modulated n- to p-type sensing transitions were observed. In the present research, p-type responses were obtained due to the higher rGO doping (1–3%). All the ZnO rods-rGO sensors were sensitive to 5–30 ppm of NO_2_. The maximum gas response was achieved at 2% rGO content (ZnO rods-2% rGO). Such gas-sensing results are not consistent with the above BET tests. This demonstrates that while the exact surface area has little bearing on NO_2_ detection, the synergistic interaction between ZnO and rGO in hybrids is critical. The three sensors’ responses versus NO_2_ concentrations and fitting curves are reported in Figure 8c. The sensor response was almost linearly dependent on NO_2_ gas concentration in the range of 5–30 ppm, implying sensor reliability.

**Figure 9:**
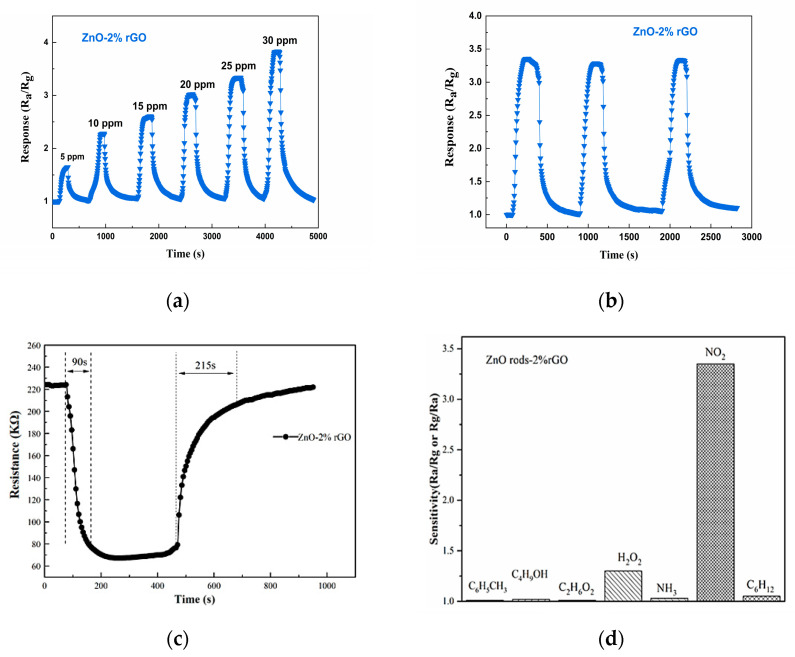
Response of the ZnO rods-2% rGO when it was in different NO_2_ gas concentrations, from 5 ppm to 30 ppm, at room temperature (25 °C) (**a**); repeatability of the ZnO rods-2% rGO when exposed to 25 ppm of NO_2_ gas 3 times, at room temperature (25 °C) (**b**); response and recovery time curves of ZnO rods-2% rGO from 25 ppm of NO_2_ (**c**); responses of the ZnO rods-2% rGO to NO_2_ and other gases (oxygen, ammonia, ethanol, toluene, n-hexane) (**d**).

In contrast to previously reported gas sensors based on ZnO rods-2% rGO, the ZnO rods-2% rGO display conventional p-type semiconductor characteristics, as shown by the data above (Figure 9). The response of the ZnO rods-2% rGO rose from 1.647 to 3.525 when the NO_2_ gas concentration increased from 5 ppm to 30 ppm. The three-cycle experiment toward 25 ppm NO_2_ at ambient temperature was performed to demonstrate the stability and reliability of the ZnO rods-2% rGO. The response and recovery times of the ZnO rods-2% rGO are 90 s and 215 s, respectively, and its gas-sensitive performance is steady and does not change visibly, with the baseline recovering to the original starting value.

**Figure 10:**
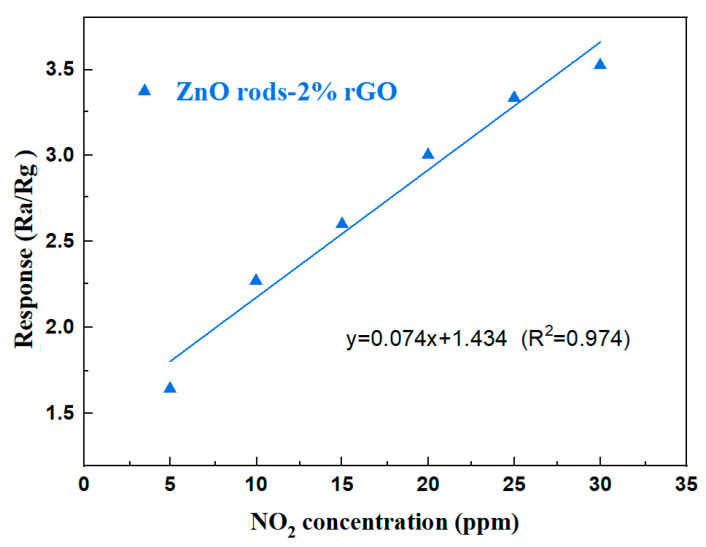
The fitting curve of ZnO rods-2% rGO to 5–30 ppm NO_2_ at room temperature.

The linear fit of ZnO rods-2% rGO has a slope of 0.074 ppm^−1^ and a correlation value of 0.974 (Figure 10). The limit of detection (LOD) was quantified using the signal-to-noise ratio approach [37]. The LOD may be determined using Formula (1):

(1) $$LOD=3\times \frac{RMS_{nosie}}{K}$$

Equation (2) was used to determine RMS_noise_ (the standard deviation of noise in the air), and it was calculated to 0.0005088 using 30 data points from the baseline of the response curve.

(2) $$RMS_{\mathrm{nosie}}=\sqrt{\frac{\sum_{i=1}^{N}{\left(R_{i}-\bar{R}\right)}^{2}}{N}}$$

And “K” is the slope of the response’s linear fitting (y = 0.074x + 1.434). The relative resistance change from the average resistance recorded can be used to calculate sensor noise. The LOD was estimated to be 20.6 ppb.

**Figure 11:**
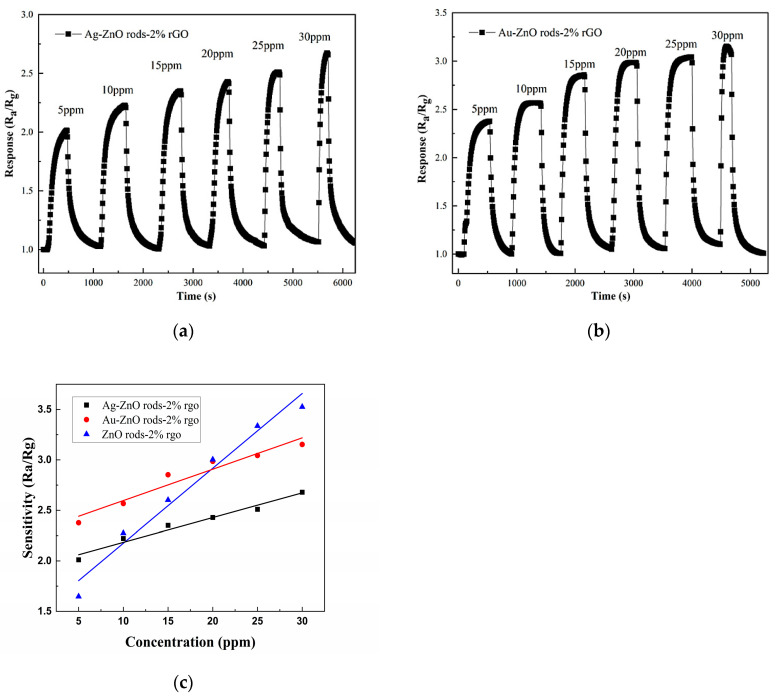
Responses of the gas sensors when they were in different NO_2_ gas concentrations, from 5 ppm to 30 ppm, at room temperature (25 °C): Ag@ZnO rods-2% rGO (**a**); Au@ZnO rods-2% rGO (**b**); ZnO rods-2% rGO, Ag@ZnO rods-2% rGO, Au@ZnO rods-2% rGO (**c**).

Showing a NO_2_ concentration range of 5 to 30 ppm, Figure 11 displays the transient response curve of the ZnO rods-2% rGO, Ag@ZnO rods-2% rGO, and Au@ZnO rods-2% rGO sensors. The S_(Ra/Rg)_ instantaneously increases when exposed to NO_2_, demonstrating the sensors’ p-type sensing properties. It is important to note that the addition of Ag and Au NPs improved the sensing of ZnO rods-2% rGO for low concentrations of NO_2_ significantly. This improvement is primarily attributable to Ag and Au NPs’ superior electrical conductivity and the creation of Schottky junctions with ZnO and rGO, which alter the materials’ natural carrier states [38,39]. Regarding this, compared with ZnO rods-2% rGO, Au@ZnO rods-2% rGO has a higher response value for NO_2_ concentrations less than 20 ppm, while ZnO rods-2% rGO shows a stronger response when NO_2_ concentration is greater than 20 ppm. Based on the aforementioned NO_2_ dynamic responses, Au@ZnO rods-2% rGO consistently exhibits a stronger NO_2_ reaction than Ag@ZnO rods-2% rGO, which may be a result of the noble metal nanoparticles’ different electrical structures.

**Figure 12:**
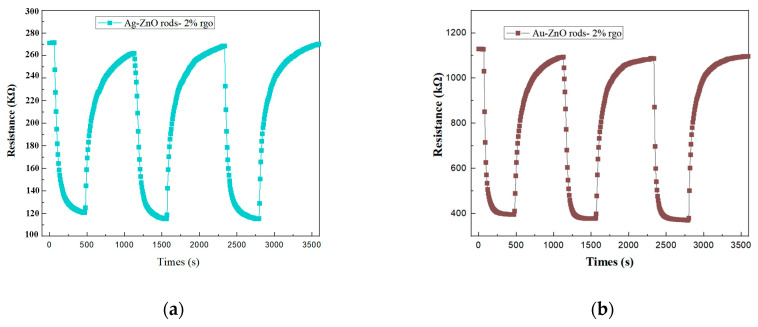
Repeatability of the Ag@ZnO rods-2% rGO (**a**) and Au@ZnO rods-2% rGO (**b**) when exposed to 15 ppm of NO_2_ gas 3 times at room temperature (25 °C).

Figure 12 shows the cyclic response curves of Ag@ZnO rods-2% rGO and Au@ZnO rods-2% rGO for 15 ppm NO_2_ gas for three consecutive cycles. From the cyclic response curves, no significant shift in the baseline was noted after three consecutive gas cycling tests.

**Figure 13:**
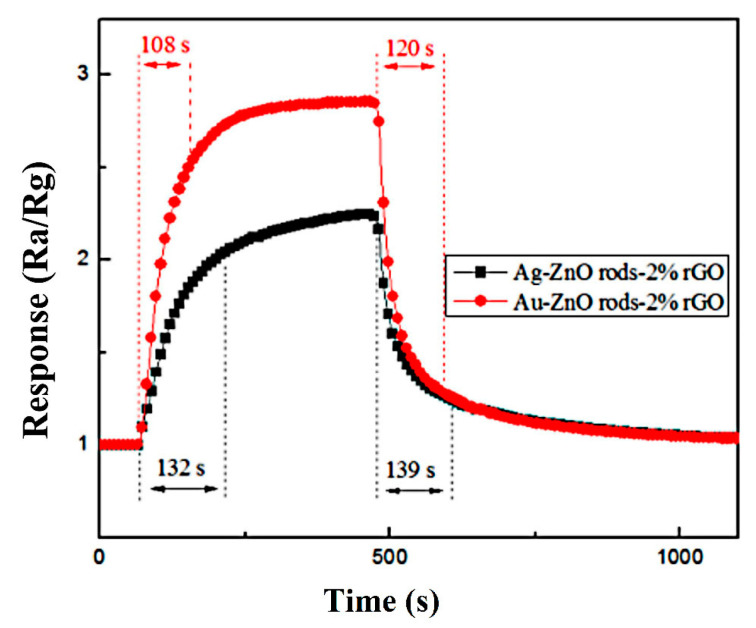
Recovery and response times of the Ag-ZnO rods-2% rGO and Au@ZnO rods-2% rGO when exposed to 15 ppm of NO_2_ gas at room temperature (25 °C).

The Ag@ZnO and Au@ZnO rod rGO response and recovery time profiles for 15 ppm NO_2_ gas are shown in Figure 13. The two sensors’ respective response and recovery times are 132 s, 139 s and 108 s, 120 s. Au@ZnO rods rods-2% rGO has better gas-sensing performance than Ag-ZnO rods-2% rGO as a whole. Compared with ZnO rods-2% rGO, the recovery times of Ag-ZnO rods-2% rGO and Au@ZnO rods-2% rGO are significantly shorter.

**Figure 14:**
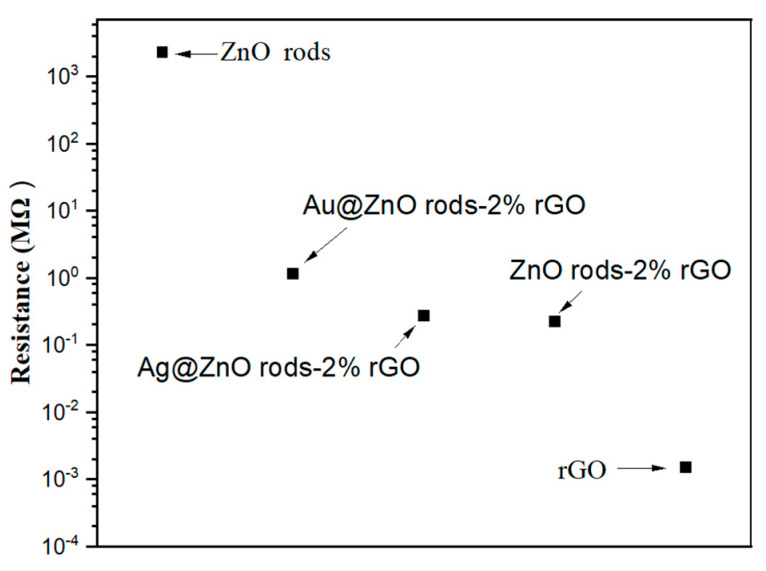
Resistance of rGO, ZnO nano rods, and ZnO/rGO composites.

The surface of the microelectrodes was coated with a material dispersion to create sensors. The resistance of the ZnO-, ZnO/rGO-, and rGO-based sensors at room temperature is shown in Figure 14. The ZnO-based sensor exhibits the highest resistance. At room temperature, it acts in an almost insulative manner. It is challenging to assess the sensing capability at room temperature because an n-type sensor exhibits an increase in resistance when exposed to NO_2_ gas. Typically, a temperature over 150 degrees is ideal for working. Due to its great electrical conductivity, rGO has the lowest resistance. The sensor based on rGO produces a poor response as a p-type sensor that shows a reduction in resistance in NO_2_ gas. Sensors based on ZnO/rGO exhibit a medium resistance with p-type sensing when ZnO and rGO are combined. ZnO rods with Au or Ag doping exhibit greater resistance than ZnO rods with 2% rGO. This is because the interfacial electron transport is hindered by the comparatively tiny (10–30 nm) size of Au or Ag NPs on the ZnO rods-2% rGO surface in Au- or Ag-doped samples.

**Figure 15:**
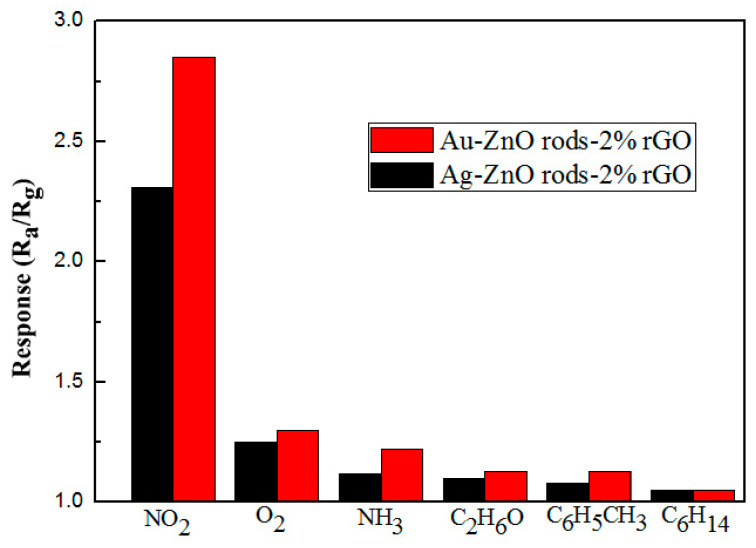
Responses of the Ag-ZnO rods-2% rGO and Au@ZnO rods-2% rGO to NO_2_ and other gases (oxygen, ammonia, ethanol, toluene, n-hexane) with 15 ppm at 25 °C.

As shown in Figure 15, we also performed electrical tests on the Ag@ZnO rods-2% rGO and Au@ZnO rods-2% rGO sensors with the same concentrations of nitrogen dioxide, oxygen, ammonia, ethanol, toluene, and n-hexane at 25 °C. These tests were performed to alleviate selectivity, another significant issue with the sensor. We discovered that the Ag@ZnO rods-2% rGO and Au@ZnO rods-2% rGO sensors have extremely good NO_2_ (2.85/2.31) selectivity, with only negligible responses to other gases.

## 4. Discussion

**Figure 16:**
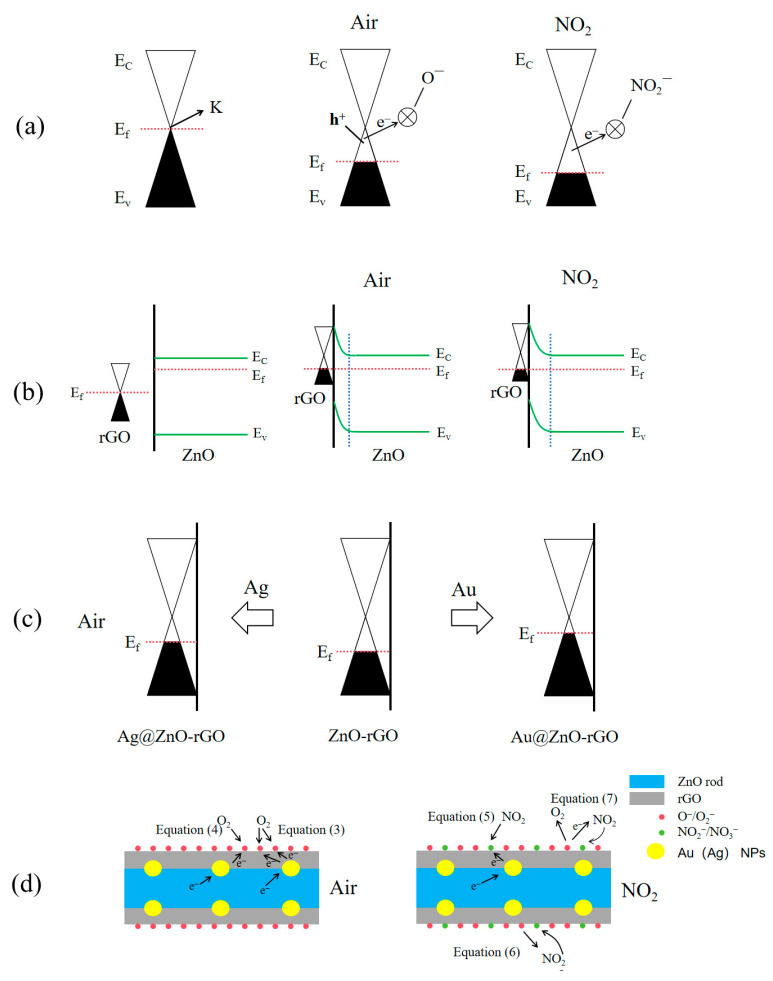
Energy band models for NO_2_-sensing mechanisms of (**a**) rGO, (**b**) ZnO rod–rGO hybrids, (**c**) Ag@ZnO rods rGO, and Au@ZnO rods rGO in air. Also shown are representative physical models for NO_2_-sensing mechanisms of (**d**) Ag (Au)@ZnO rods-2% rGO.

The findings reveal that pure rGO exhibits increased p-type electrical conductivity and NO_2_ responsiveness with graphene loading at the optimal amount of 2 wt% while undoped ZnO exhibits n-type gas-sensing characteristics. Due to its chemical reduction-based synthesis and electron-withdrawing oxygen functional groups, rGO exhibits p-type semiconductor properties. Figure 16a’s band diagram can be used to describe the p-type NO_2_-sensing mechanism of rGO. When rGO is exposed to air at a moderate temperature, oxygen molecules chemisorbed on the surface form oxygen species (O_2_^−^, O^−^) (Equations (3) and (4)), which simultaneously induce holes (h^+^) and positive charge carriers, and remove some electrons from the valence band, converting graphene into a p-type metal with a Fermi level below the K-point. As a result of being exposed to NO_2_, molecules of NO_2_ are chemisorbed into NO_2_^−^ ions (Equation (5)), which further remove electrons from the valence band and increase the concentration of holes, causing the resistance to drop [40].

O_2_(g) + 2e^−^ → 2O^−^(ads) (3)

O_2_(g) + e^−^ → O_2_^−^(ads) (4)

NO_2_(g) + e^−^ → NO_2_^−^(ads) (5)

NO_2_(ads) + O^−^(ads) → NO_3_^−^(ads) (6)

NO_2_(ads) + O_2_^−^(ads) → NO_2_^−^(ads) + O_2_(g) (7)

Due to the difference in work functions, when rGO comes into contact with ZnO (Figure 16b), the electron moves from n-type ZnO to p-type rGO. This decreases the carrier concentration (electron/hole) and raises the resistance of ZnO/rGOs. In addition, an electrical field develops within. In the presence of air, oxygen molecules adsorb on the ZnO/rGO surfaces and are ionized to form oxygen ions. These oxygen ions then draw electrons from the ZnO conduction band, resulting in an expansion of the ZnO electron depletion layer and upward band bending. When ZnO/rGOs is exposed to NO_2_, an electron moves from there to the adsorbed NO_2_. Since NO_2_^−^(ads) has a higher electronegativity than O_2_^−^ (ads), they combine to generate NO_3_^−^(ads) (Equation (6)). The internal electrical field of ZnO is weakened as a result of the reduction in free electrons, which increases the gap between E_c_ and E_f_. In the presence of NO_2_, the electrons in rGO are transported to ZnO, increasing rGO’s hole content. They therefore have superior NO_2_-sensing capabilities (S = R_a_/R_g_).

Electron sensitization caused by the interfacial electron transition between Ag (Au) nanoparticles and ZnO-rGOs has a significant impact on the hole accumulation layer for Ag@ZnO rods and Au@ZnO rods with 2% rGO. After contact, the Fermi levels of Ag (Au) nanoparticles and ZnO-rGOs are balanced at the Ag/ZnO-rGO interface due to the different work functions of the two materials (Figure 16c). At the interface of Ag (Au) nanoparticles and p-type ZnO-rGOs, electrons in Ag (Au) nanoparticles are injected into the valence bands of ZnO-rGOs. In this manner, the Fermi energy of ZnO-rGOs rises while the Fermi energy of Ag (Au) falls until they are equal. This electron transfer between Ag (Au) nanoparticles and ZnO-rGOs is confirmed by the increased Rg of Ag (Au)-anchored ZnO-rGOs sensors in Figure 14. As more electrons are snatched from the conduction band when the Ag (Au)-anchored ZnO-rGO sensor is exposed to NO_2_, the thickness of the electron depletion layer increases significantly, and the resistance value of the sensor alters more noticeably, showing a p-type response (Figure 16d). Au@ZnO rods-2% rGO shows a higher R_g_ than Ag@ZnO rods-2% rGO, implying that more electrons were injected into the valence band. As a result, the magnitude of the decrease in resistance in Au@ZnO rods-2% rGO is much larger than that in Ag@ZnO rods-2% rGO. As shown in Figure 11c, the response of Au@ZnO rods-2% rGO is higher than that of Ag@ZnO rods-2% rGO under the same conditions.

Both chemisorbed oxygen and the catalytic additive in the materials affect the sensing performance. As catalysts, Au (Ag) NPs can not only provide abundant active adsorption sites on the semiconductor surface to promote the reaction between oxygen adsorption and NO_2_, but also reduce the activation energy of the reaction and accelerate the reaction. As shown in Figure 11c, Au@ZnO rods-2% rGO shows a greater response than ZnO rods-2% rGO when NO_2_ concentration is below 20 ppm. However, ZnO rods-2% rGO shows a stronger response than Au@ZnO rods-2% rGO when NO_2_ concentration is greater than 20 ppm. This may be related to the chemisorbed oxygen (Oc) on the surface, as the adsorbed oxygen content in ZnO rods-2% rGO is relatively high (Figure 6g), speeding the interaction between the deposited oxygen ions and NO_2_ molecules (Equation (7)), exacerbating the change in resistance, and improving the sensitivity of the sensor toward higher NO_2_ concentration.

In short, the outstanding sensing characteristics of Au (Ag)@ZnO rods-rGO hybrids were accomplished through the synergistic impact of ZnO/rGO hybrids and Au (Ag) NPs [26,41].

## 5. Conclusions

In summary, we succeeded in effectively producing ZnO rods-rGO gas sensors decorated with Au NPs and Ag NPs using a simple hydrothermal process. First, we tried to adjust the additional content of rGO. The ZnO rods-2% rGO sensor had the greatest gas response of 3.5 toward 30 ppm of NO_2_ gas at room temperature among the ZnO rods-rGO hybrids. The inclusion of Au NPs and Ag NPs improves the gas-sensing property of ZnO rods-2% rGO even further. The Au@ZnO rods-2% rGO sensor has a maximum response of 2.8 to 15 ppm of NO_2_ gas at ambient temperature, with response and recovery times of 108 and 120 s, respectively, and no deviation in the initial value after recovery. The synergistic impact of the ZnO rods and rGO, the catalytic capability of Au (Ag) NPs, and the large specific surface area all contribute to the sensors’ improved gas-sensing performance. These ternary hybrids are capable of detecting extremely low NO_2_ concentrations with high accuracy.

## Data Availability

The data presented in this study are available on request from the corresponding author.
